# Children's use of race and gender as cues to social status

**DOI:** 10.1371/journal.pone.0234398

**Published:** 2020-06-22

**Authors:** Tara M. Mandalaywala, Christine Tai, Marjorie Rhodes

**Affiliations:** 1 Department of Psychological and Brain Sciences, University of Massachusetts Amherst, Amherst, Massachusetts, United States of America; 2 Department of Psychology, University of Hawai‘i at Mānoa, Honolulu, Hawaii, United States of America; 3 Department of Psychology, New York University, New York City, New York, United States of America; University of Maryland, UNITED STATES

## Abstract

Social hierarchies are ubiquitous and determine a range of developmental outcomes, yet little is known about when children develop beliefs about status hierarchies in their communities. The present studies (3.5–6.9 years; *N* = 420) found that children begin to use gender and race as cues to status in early childhood, but that gender and race related to different status dimensions and had different consequences for inter-group attitudes. Children expected boys to hold higher status as defined by access to resources and decision-making power (e.g., having more toys and choosing what other people play with) but did not expect boys to have more wealth overall. Gender-related status beliefs did not relate to gender-related social preferences; instead, children preferred members of their own gender, regardless of their status beliefs. In contrast, children expected White people to be wealthier than Black people, and among some populations of children, the belief that White people were higher status (as defined by access to resources and decision-making power) weakly related to pro-White bias. Children’s status-expectations about others were unrelated to beliefs about their own status, suggesting children more readily apply category-based status beliefs to others than to themselves.

## Introduction

Status hierarchies are ubiquitous across diverse human cultures and many animal species [1], and have a range of personal and group-level implications [2,3]. Status hierarchies can be based on a wide variety of factors, including (but not limited to) physical size or prowess, access to resources, wealth-holding, social or decision-making power, and prestige or social influence (see [4,5] for reviews). Despite the fact that social status can have this multi-dimensional nature, comprising asymmetric relations along one or more of the aforementioned dimensions, the ability to detect and track status hierarchies emerges early in human development. For example, infants use cues such as physical size and group size to predict dominance relations [6,7,8], and preschool-aged children show sensitivity to a broad set of cues to status (e.g., [9,10,11,12,13]) and use these cues to predict a range of behaviors. For example, 4-year-olds expect resource-rich (high-status) individuals to share more than resource-poor (low-status) individuals [14].

Whereas previous work has examined how infants and children infer status differences between people when given clear status cues (e.g., children can infer who is dominant when told about differences in physical size or access to resources), less is known about the developmental trajectory of children’s beliefs about status differences between social groups that children are likely to encounter in their communities. In particular, we investigated whether children use a person’s gender (Study 1) or race (Study 2) as a cue to their social status—relevant domains given the persistent gender [15,16] and racial [17,18] inequalities in social power and access to resources found in society today.

Across two studies and in a diverse group of children, we asked: (1) when do children begin to use gender or race as a cue to a person’s social status, (2) how do beliefs about status shape children’s inter-group attitudes and preferences, and (3) how do children view their own, subjective, status? These questions are important because group-based beliefs about status (e.g., using race or gender to infer relative position in a social hierarchy) have been implicated in the development of negative attitudes towards members of groups that are perceived as low status [19, 20] and the stigmatization and devaluation of members of low-status groups (e.g., [21]). Believing certain groups to be lower status also leads members of stereotypically lower-status groups to feel that their opportunities are limited or undesirable (e.g., [22,23]).

## The development of group-based beliefs about status

### Social status and race

In cultures where race and socioeconomic status covary, children often begin to use race to predict social status during early childhood (e.g., [24,25]). This research has most closely examined the links between race and prestige [22] and between race and wealth [26,27,28]. In terms of prestige, 6-year-old children in the United Sates expect Black people to have lower-status occupations than White people, and even assume that novel occupations are lower status when they are described as held by Black, rather than White, people [22]. In terms of wealth, preschool-aged children expect White people to have nicer-looking possessions and houses than Black people in both the United States [26,28] and South Africa [27]. These studies included White and Black children, and generally found that participant race did not affect whether children associated race and prestige, or race and wealth (but see [28] where White preschoolers held stronger associations between race and wealth than did Black preschoolers). Because both White and Black children showed the same pattern (associating White, and not Black, people with wealth and prestige), children’s beliefs about how race and status covary appear to reflect abstract beliefs about societal structure, rather than general in-group preferences (in which case Black children would have been more likely to associate Black, rather than White, people with wealth and prestige).

### Social status and gender

Among adults, gender is another robust cue to status, however, the extent to which children view status as tied to gender has rarely been examined. Given persistent gender inequality in both social power [15] and resource-holding [16], and because children often view gender as a highly informative social characteristic [29,30], it is possible that children link gender to status. The data supporting this idea are mixed. Children as young as three- and four-years-old use features often associated with men (e.g., cues to physical strength, masculine facial characteristics) to infer who is in charge [31]. Additionally, when researchers explored whether children use gender as a marker of occupational prestige, one dimension of status, they found that 6- to 8-year-olds in the United States viewed more masculine jobs as higher status than more feminine jobs [23]. This result could be explained by the fact that children are statistically more likely to see men in prestigious, high-power positions than they are to see women in comparable positions (e.g., male principals vs. female teachers). In contrast, although Olson and colleagues [27] found that children in South Africa used race to predict who had nicer possessions, these same children did not have similar intuitions for gender. This is perhaps unsurprising; although persistent pay discrepancies exist between males and females [16], wealth differences by gender might not be salient to children, particularly those growing up in heterosexual two-parent households where income is combined across the family.

Another reason for inconsistent findings is that children’s status beliefs are likely to interact with—and perhaps come into conflict with—other aspects of their gender-related beliefs. For girls in particular, the belief that boys and men are higher status goes against their own tendency to prefer their gender in-group, a tendency that is often strong in early childhood (i.e., [30,32]). A deeper dive into Liben, Bigler, & Krough’s study [23] of the gender stereotypes around occupational prestige provides some support for this idea. Although both male and female participants in this study rated masculine jobs as higher status, the difference in ratings of masculine and feminine jobs were significantly greater among male participants than among female participants. This suggests that although cultural input and messages can begin to overwhelm gender in-group bias, these messages are unlikely to erase completely responses that are rooted in an in-group bias.

Although robust in-group biases might make it difficult to find evidence that children use gender as a marker of status, under certain conditions we might be more likely to detect evidence for gendered beliefs about status. Given the types of input children regularly receive, we predict that when status is defined in terms of social power, we might be *most* likely to detect gendered beliefs about status. For example, factors such as the content of children’s media may lead children to be more likely to link gender and status based on social power (at least more so than gender and status based on wealth). Children’s storybooks and popular movies often depict girls and women as helpless and submissive, but boys and men as more skilled and in decision-making roles [33,34,35]. However, although studies provide robust evidence that children display awareness of traditional gender roles from as young as 2 years of age [36,37,38], whether children use gender as a marker of status when given information about social power has not previously been examined.

### Measuring group-based beliefs about status

Prior studies investigating children’s use of race and gender as a cue to a person’s social status suggest that different dimensions of status (e.g., wealth, social power, social influence, prestige, etc.) may be more closely related to certain social domains than others. For example, wealth appears to be more tightly linked to race than to gender (e.g., [27]). However, traditional methods of studying status beliefs in children have typically used tasks that are specific to a particular status dimension. For instance, researchers studying wealth-based status would use a wealth-matching task, where children are shown possessions (e.g., cars, houses, toys) that vary in quality or quantity and asked to match each target individual to the possession they go with. This approach is useful, but also limits our ability to compare how children use different social domains (e.g., gender or race) to make predictions about the status of others across different dimensions of status. To overcome this limitation, we developed a flexible new task that allows multiple dimensions of status to be presented within the same experimental paradigm, as opposed to the traditional method of using different tasks to tap into different dimensions of status. In the present study, we used this new task (i.e., the rope task) to examine beliefs about status that incorporated both wealth *and* social power. As used across the two studies presented here, the rope task did not permit us to determine whether children relied more on wealth *or* social power cues (or equally on both) when making inferences about status. However, using this task allowed us to compare directly children’s use of race and gender as cues to these dimensions of status within a single experimental paradigm. This approach both reduces the risk that task-specific features lead to different results across different social domains, and helps us begin to clarify similarities or differences in the developmental trajectories of children’s reasoning about race- and gender-based status.

Additionally, we compared children’s responses on this new task to their responses on a task that has been used in previous research, where status was presented in terms of wealth-based cues only (e.g., the wealth-matching task [27,28]), to explore how responses on the rope task compared to responses on an established task. Although the studies presented here were not explicitly designed to assess whether children’s beliefs about gender-based status relied on different cues (e.g., wealth, social power, etc.) than their beliefs about race-based status, comparing children’s responses across the two tasks could shed light on this question. For example, if children use gender to predict status on the rope task (where wealth- and social power-based cues are presented) but not on the wealth-matching task (where only wealth-based cues are presented), this would suggest that children rely more heavily on cues to social power than on cues to wealth when inferring one’s status based on gender. Future research can and should use the rope task to assess how each dimension of status relates to different social domains, an idea we return to in the Discussion, and the studies presented here serve the purpose of introducing this new tool that can more precisely examine these questions in the future.

### The consequences of status beliefs

Once status beliefs form, they could have a wide variety of consequences, both positive and negative. For instance, by drawing attention to inequality, status beliefs might foster support for equity-enhancing policies (a pattern found in adults: [39,40]). However, group-based beliefs about status have also been hypothesized to be foundational in the development of social bias, leading people to dislike lower-status groups [19,20]. Consistent with the idea that status beliefs are likely to affect social preferences and behaviors, 4-years-olds prefer people associated with high-wealth (versus low-wealth) items [28], and children who demonstrate pro-wealth preferences also show an implicit pro-White preference, both in the United States [41], and in South Africa [42] (but see also [43] for no relation). Even children as young as 21 months of age show preferences for socially dominant actors, at least in cases where social dominance is directly portrayed in the experiment itself (e.g., by observing the interactions of two actors: [44]) and where dominance does not need to be inferred based on membership in real-world groups (as we tested here). Unfortunately, empirical data directly testing whether status beliefs about real-world groups are directly related to social biases are lacking. In the only study we are aware of, Olson et al. [27] found that South African children (ages 3–10) who associated White people with wealth were only marginally more likely to prefer a White social partner, providing inconclusive evidence that status beliefs and bias development are strongly related. Here, we build on this previous work to test whether the relation between race-based status beliefs and the emergence of racial bias is robust across various measures and dimensions of status among children growing up in the United States. We also test whether gendered beliefs about status relate to variation in the development of gender-based social preferences, a question not previously explored. By studying the relations between status beliefs and social biases across social domains, we can begin to determine whether status beliefs are necessary for the development of bias across social domains (as previously hypothesized [19]) or whether status beliefs are only necessary for certain types of bias (e.g., necessary for racial, but not gender, bias).

### Subjective status in children

Holding associations between status and race or gender can have implications for more than just inter-group attitudes; these beliefs may also affect the way children view themselves and their beliefs about the opportunities available to them. For example, African-American children from low socioeconomic status (SES) backgrounds have been found to be less likely to aspire to occupations considered to be prestigious than children from higher SES backgrounds [22]. This is not to negate the fact that structural barriers based on discriminatory policies lead to inequalities between social groups. However, previous studies find that, among adults, one’s *subjective* social status (i.e., their perception of their own status) is a better predictor of their outcomes, including well-being and longevity, than their objective social status (i.e., [45,46]). Studies in children and adults suggest that perceptions of one’s place in societal hierarchies can affect outcomes across a variety of important domains (e.g., from educational and occupational aspirations to health and well-being) over and above their actual socioeconomic status. Therefore, it is important to understand whether and when children begin to apply group-based beliefs about status to evaluate their own position in the social hierarchy. Subjective status has only recently been studied in children, and research suggests that by 10 years of age, children’s subjective status assessments begin to match family SES [47], with recent evidence suggesting that children as young as 3-years-old also demonstrate some awareness of family-based wealth [48]. Here, we also test children’s perceptions of their own social position, and whether their beliefs about themselves relate to their more generalized beliefs about how gender and race predict social status.

### Overview

In sum, across two studies and a diverse sample of children ages 3.5–6.9-years-old, the present research tested three phenomena surrounding the development and consequences of children’s status beliefs. Children at these ages often show pro-White biases, and have been found in some studies to use race to predict wealth-based social status [27,28], making it an appropriate age range to test children’s beliefs across multiple social domains and multiple dimensions of social status.

First, we examined the emergence of tendencies to use social domains, in particular whether children at these ages used gender (Study 1) or race (Study 2), to predict social status. Although children at these ages often use race to predict wealth-based social status (i.e., saying that White children live in nicer houses than Black children), they have not been shown to use gender to predict wealth-based status (i.e., being *equally* likely to say that girls and boys live in nicer houses). Given the lack of reliable correlation between gender and wealth among children living in heterosexual, two-parent households, we thought that children might be more likely to use gender as a cue to status when given additional cues to status, such as differences in social power. Thus, in Study 1 we predicted that children might be more likely to use gender as a cue to status on the rope task, where multiple cues to status were presented, than on a task where only cues to wealth were presented. We did not make a similar prediction for Study 2, when children were asked about race, as information about wealth was presented in both status tasks.

Second, we examined whether children’s gender or racial preferences were predicted by their belief that gender or race is a cue to social status. Given the strong gender in-group biases that children at these ages show [20], in Study 1 we did not predict that children’s status beliefs about gender would predict their gender preferences. In contrast, theoretical models of the development of racial prejudice hypothesize that status beliefs lay the groundwork for anti-Black bias [19], and previous work has shown a weak relation between anti-Black prejudice and children’s belief that White people are wealthier than Black people [27]. Therefore, in Study 2, we predicted that children who used race as a cue to status would show anti-Black prejudice in their social affiliation decisions.

Third, we examined the relation of children’s gender- and race-based status beliefs to beliefs about their own personal status. We made no predictions about children’s beliefs about their own status. By 10-years-old, children’s evaluations of their own status correlates with family income [47], and even young children are capable of assessing family wealth generally and in broad strokes [48]; however, children also hold positive views of themselves [49] that might impede their ability to evaluate their own status.

We examined these three phenomena in a diverse group of participants. By including participants from a diverse range of racial-ethnic backgrounds, as well as equal numbers of male and female participants, these studies allowed us to examine how children’s own group memberships (and any in-group biases that might accompany group membership) affect the development, expression, and application of status beliefs. Including a diverse sample of participants is especially important in the domain of status hierarchies for several reasons. First, there is some evidence that the development of status beliefs varies across members of different racial-ethnic groups [28]. Second, beliefs and attitudes about status hierarchies are often invoked as explanations for why individuals from marginalized or lower-status groups sometimes accept, rather than reject, hierarchies that systemically disadvantage them (e.g., [19,50]), making it crucial to include participants from stigmatized groups. Finally, because children’s beliefs and preferences surrounding race and gender often differ at these ages (e.g., [20,51]), we examined these two social dimensions in separate studies, focusing on gender in Study 1 and race in Study 2. Both studies were approved by the Institutional Review Board at New York University (approval number: IRB-FY2016-760).

## Study 1

### Method

#### Participants and procedures

Participants were recruited and tested at the Children’s Museum of Manhattan (CMOM)—a private, not-for-profit museum located in a large, urban city (pop. ~ 8.5 mil, median household income = 60,752; percent of population living below the poverty line = 18.9%: [52])—in a single 5–15 minute research session conducted between September 2016 and July 2018. Written parental consent was obtained for all participants and children provided oral assent. In an attempt to collect data from a racially and socio-economically diverse group of participants, we collected data during both pay-to-enter days, as well as monthly free-admission days. Participants were a diverse group of children ages 3.5–6.9 (*M*_*age*_ = 5.01; *N* = 215; 48.8% female; White: 33.0%; Hispanic: 13.0%; Asian: 16.7%; Black or African-American: 10.7%; Multiracial: 14.9%; other: 1.9%; not provided: 9.8%). Child race was determined by parental report. We were unable to collect household income information from parents and guardians; however, slightly less than half of parents (48%) provided employment or education information and among this subset, 90% had at least one-parent that was employed full-time and/or who had a college degree.

G*Power calculations (with 80% power) based on an effect size of 0.29 (based on a pilot study of the rope task: [53]) indicated the need for approximately 95 male and 95 female participants to be sufficiently powered to detected an interaction of participant gender (male; female) and target gender (boy; girl). An ability to detect this interaction is important given robust own-gender biases often expressed in children in our age range [20]. We completed data collection on the weekend we reached the pre-determined sample size, leading to slightly more participants than planned, and we have reported all exclusions and the rationale for exclusion. We pre-registered this study (including the study design, hypotheses, predicted patterns, and planned analyses) on Open Science Framework (https://osf.io/c94v3).

Children completed two tasks to assess their status beliefs. Children first completed the “rope task,” a new task (adapted from subjective status ladders used in adults, see [54]) that provided a measure of children’s tendencies to use gender as a cue to social status when status was defined in terms of social power *and* wealth. Children next completed the “wealth-matching task,” an established task that provided a measure of children’s tendencies use gender as a cue to social status when status was defined in terms of wealth-holding alone (e.g., [28]). To determine the consequences of children’s beliefs about the covariance of gender and status, children then completed a “social preferences task,” that measured whether children prefer to associate with children of a particular gender. Participants also completed a brief “wealth-preferences task” (e.g., do children prefer targets associated with wealth, regardless of their gender? see [27]), after the wealth-matching task, and before the social preferences task. Because the wealth-preferences task was not relevant for our primary research questions, we do not include these analyses in main text, but include them in the SOM. With the exception of stimuli for the social preferences task, in which images were taken from a Google search, all stimuli were images of children making a closed-mouth neutral-face taken from the Child Affective Facial Expression (CAFE) set [55,56] that had been identified by their parents as Hispanic. Children at these ages tend to rely on differences in skin color, rather than physiognomy, to infer racial group membership [57]; therefore, we chose to use images of Hispanic children—all similar in skin tone—as stimuli across all tasks in Study 1 to avoid giving children obvious cues to race and instead tacitly encouraging them to focus on gender as the salient social dimension. For authorized Databrary users, the full stimulus set can be viewed at: https://nyu.databrary.org/volume/599. At no point during the task were any of the stimuli labeled by gender or referred to using gendered pronouns, enabling us to assess whether children attended to gender spontaneously.

#### The rope task

**Fig 1:**
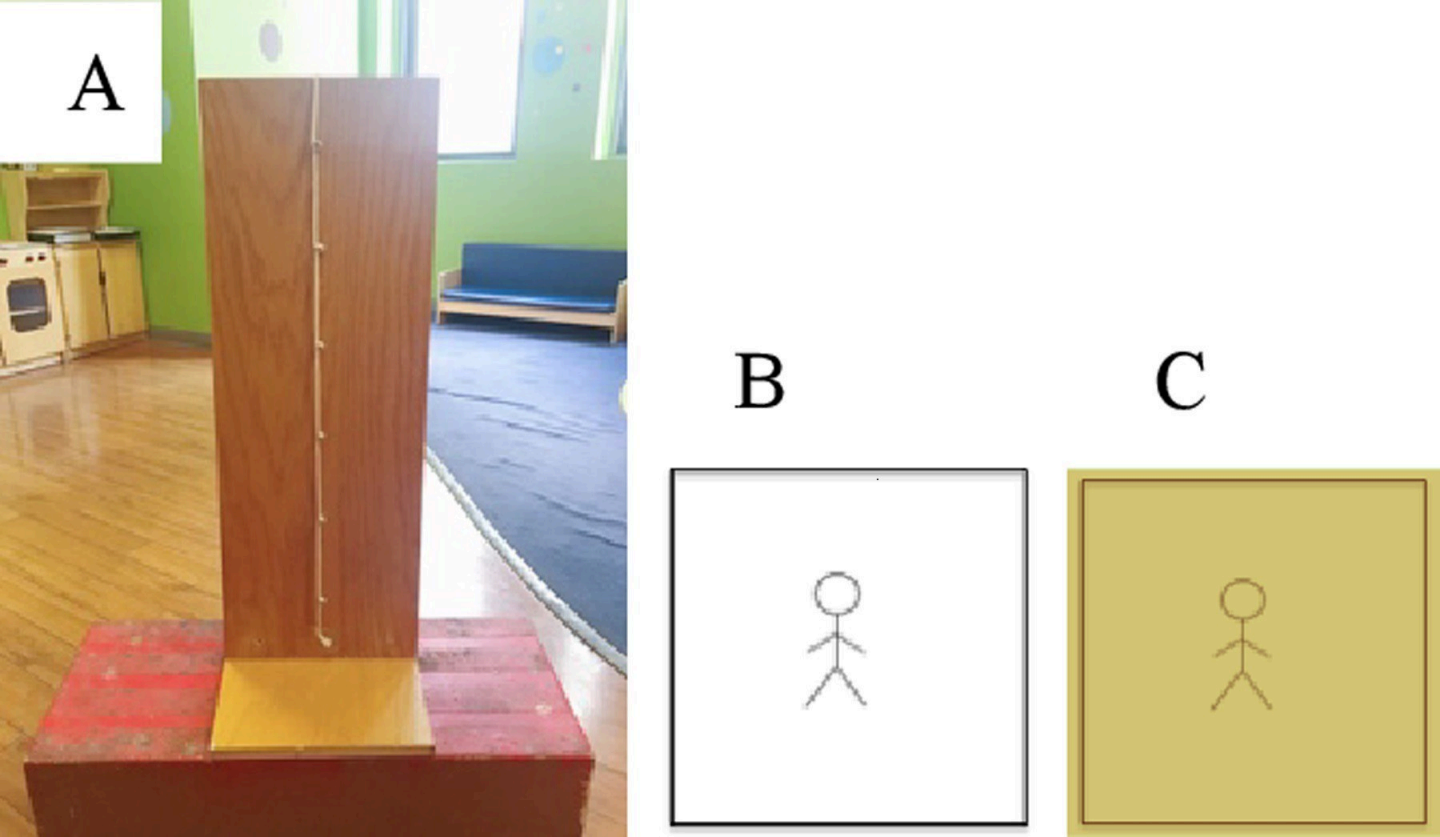
Pictures of (A) board with rope and pegs used for the rope task, including (B) comprehension check stimulus, and (C) example of subjective status card stimulus.

Children were presented with a 13 x 35 inch wooden board with six equidistant pegs placed in a column along the front of the board. A white rope ran vertically down the board, wrapping around each peg (Fig 1A). Participants were first trained on the use of the board. In particular, they were told, “Kids at the top of the rope [*experimenter gestures to the top peg on the board*] have lots of toys and new clothes, and they always get to pick the games that everyone else plays at recess and the snacks that everyone else eats at snack time. Kids at the bottom of the rope [*experimenter gestures to the bottom peg on the board*] don’t have any toys or new clothes, and they never get to pick the games that anyone plays at recess or the snacks that anyone eats at snacktime. But you know what? Kids don’t have to go just at the top [*experimenter gestures at the top peg again*] or the bottom [*experimenter gestures at the bottom peg again*]. They can go at any of these places in the middle too [*experimenter gestures at all middle pegs on the board*, *moving from the bottom to the top*].” After training, participants were tested on their comprehension. Participants were presented a small 2 x 2 in laminated card that depicted a non-gendered stick figure on a white background that had a small hole at the top that could be used to hang the card on the selected peg (Fig 1B). Participants were told that, “This kid has lots of toys and lots of new clothes and always gets to pick the games and the snacks. Can you put this kid on the rope where they go?” If they selected the correct response (putting the target card on the top peg), they were told that was correct and asked the next comprehension question. If they selected the incorrect response (putting the target card on any peg except for the top peg), they were given corrective feedback (i.e., “That’s a good idea, but remember…”) and asked the question again. This same procedure was repeated for a low-status target card (where the correct response was to put the target card on the bottom peg), and for a middle-status target card (where the correct response was to put the target card on any of the middle pegs, but not on the top or bottom peg). The full script for all measures can be found at: https://osf.io/29vcu/?view_only=. Participants were asked each comprehension question until they gave the correct response, or a maximum of three times, whichever occurred first.

After completing the comprehension check questions, participants were asked to select a laminated card to represent them. These cards had a non-gendered stick figure on a color background (purple, yellow, green, or orange; children could choose their preferred background, see Fig 1C for an example). To assess participants’ subjective status beliefs, participants were then asked, “Can you put yourself on the rope where you think you go?” Participants’ response on a scale from 1 (lowest status) to 6 (highest status) was recorded. Finally, participants completed two critical trials, in which they were asked to place a (stereotypically high-status) boy target and a (stereotypically low-status) girl target on the rope. To complete these trials, participants were presented with two laminated cards, one with an image of a boy and the other an image of a girl [placed horizontally side by side]. Participants were presented with both cards at the same time, as the experimenter said, “Here are two kids. We’re going to put each of these kids on the rope, one at a time”. Working from left to right, the experimenter pointed to a specific card and asked the participant, “Can you put this kid on the rope where you think they go?” Participants could respond between 1 (lowest status) and 6 (highest status) for each target. Although participants could see the both stimuli prior to making their first decision, the order in which participants were asked to make their decisions was counterbalanced across participants. Roughly half of participants were asked to put the boy on the rope first, and the other half of participants were asked to put the girl on the rope first. We removed the first target after the participant made their decision so that the full rope was available to the participant for both targets, meaning that participants could choose to put targets on different pegs or on the same peg. There were no effects of presentation order; therefore, data were collapsed across presentation orders in all analyses. An example video of the rope task being administered can be found on Databrary (https://nyu.databrary.org/volume/599).

#### Wealth-matching task

In a task adapted from Shutts et al. [28] and Olson et al. [27], participants were presented with a laminated page, on which the images of two houses were presented side by side. These houses were matched in many characteristics, such as general size and color; however, one of the houses appeared well-maintained and the other house appeared more run-down. Children in this age range readily infer wealth from these types of house cues [58, see also 27,28]. Participants were directed to look at these houses by the experimenter, who said, “See this house? And see this house?” (*gesturing first to the left and then the right house*). The experimenter then placed two laminated cards, each depicting an individual child, one boy and one girl, directly below and in-between these houses. Participant attention was drawn to these target children by the experimenter saying, “See this kid? And see this kid?” The experimenter then asked the critical question, “Can you put each kid in the house they live in?” Participants then put the laminated card of each child on top of the house they thought that child lived in. If the participant did not put both cards on top of a house, the experimenter pointed to the remaining card and said, “And can you put this kid in the house they live in?” Participants could put both target cards on top of the same house; however, no participants did this. Stereotypical responses (i.e., putting the boy in the nicer house and the girl in the less nice house) were scored as “1” and counter-stereotypical responses were scored as “0”. Participants completed two trials of this task, where the only thing that varied between trial one and trial two was the order in which the target child cards were presented below the houses (i.e., in trial one, the boy was on the left and the girl was on the right, and vice versa for trial two). Although participants completed two trials, we found order effects on this task that made it difficult to interpret responses to the second trial; therefore, we report results for the first trial only (see S1 File–SOM–for more details).

#### Social preferences: Preference for male or female targets

Finally, participants completed a forced choice social preference task where they were presented with side by side pictures of a boy and a girl. Participants were told, “Here are two kids. Who do you want to invite to your birthday party?” Participants completed three trials of this task, and were presented with new pairs of stimuli and a new activity each time (e.g., “Who do you want to play with at the park? Who do you want to invite to the movies with you?”). Choosing the boy (high-status) target was scored as “1” and choosing the girl (low-status) target was scored as “0”.

#### Analysis plan

Data were analyzed in R using the lme4 package [59], specifying either a Gaussian (rope task, self status) or binomial (wealth-matching, social preference) distribution. For all analyses where each participant contributed more than a single data point (i.e., for the rope task and the social preference analyses), we included participant id as a random effect to avoid pseudo-replication. Because the relevant social dimension in Study 1 was gender, and in the United States, males frequently hold more resources and power than females (e.g., [15,16]), target gender and participant gender were treated as proxies for social status, with boy targets and male participants considered “high-status” and girl targets and female participants considered “low-status.”

*Analyses of children’s use of gender as a cue to social status*. For the rope task, participants who failed to pass the comprehension check for either the high-status or low-status comprehension cards were excluded from analyses (excluded *n* = 10; final *N* = 195). Because effects might vary as a function of participant’s membership in a gender group that is stereotyped as high or low in status, our primary analysis examined the main and interactive effects of target gender (boy vs. girl), age (as a continuous, mean-centered variable), and participant gender (male participants vs. female participants). As described in more detail in the SOM, this was a slight deviation from our pre-registered analysis plan.

For the wealth-matching task, we first examined whether the probability that participants responded in a stereotypical manner (i.e., saying the boy lived in the nicer house) differed from chance. We next examined whether the probability of responding in a stereotypical manner varied across age or as a function of participant gender (age x participant gender).

Finally, to compare responses across the two status tasks, we created a variable (response type) based on whether children used gender as a cue to status in a stereotypical or counter-stereotypical way (as measured by either the rope task or the wealth-matching task: see Table 1 in Results). On the wealth-matching task, children could only respond in a stereotypical (placing the boy in the nicer house and the girl in the less nice house) or a counter-stereotypical (placing the girl in the nicer house and the boy in the less nice house) manner. On the rope task, children could respond in a stereotypical manner (placing the boy in a higher position than the girl), a counter-stereotypical manner (placing the girl in a higher position than the boy), or in an equal manner (placing both the boy and the girl in the same position). We then examined whether response type on the rope task predicted response on the wealth-matching task, and whether this relation changed with age (response type on rope task x age).

**Table 1:** Number of Study 1 participants who provided a given response type for the rope task and wealth-matching task, separately for male and female participants. Table includes only participants whose parents reported child gender (*n* = 195).

	*Response type on Rope task*		
	*Equal*	*Stereotypical*	*Counter-stereotypical*
**Rope task**	Boy & girl in same position	Boy in higher position than girl	Girl in higher position than boy
Female participants	*n* = 13	*n* = 48	*n* = 47
Male participants	*n* = 7	*n* = 57	*n* = 23
**Wealth-matching task**	n/a	Boy in nicer house	Girl in nicer house
Female participants		*n* = 48	*n* = 66
Male participants		*n* = 51	*n* = 40

*Analyses about the consequences of gender-based status beliefs*. Our primary analysis tested for the main and interactive effects of response type on the rope task (stereotypical vs. counter-stereotypical), age, and participant gender on participant’s gender preferences. Due to the small number of participants who responded in an “equal” manner on the status task, for whom we also had participant gender data, in this latter model (where participant gender and response type were both included) we excluded “equal” responders from analyses (for these analyses, *n* = 175). We then repeated the above analyses, substituting response type on the wealth-matching task for response type on the rope task, to examine whether one type of status task better predicted gender preferences.

*Analyses about subjective status*. Because participant gender (and thus membership in a gender group that is stereotyped as higher or lower in status) might affect participant’s ratings of their own status, we first examined the main and interactive effects of age and participant gender on participant’s subjective status rating. We then added response type on the rope task to this model, to examine the relative contribution of participant’s gender-based status beliefs on their subjective status ratings.

As a final note, we completed the analyses as indicated in the pre-registration, with four exceptions. These exceptions are described in full in the SOM. For all analyses reported in the main text and SOM, data and analytic code across are available at OSF: https://osf.io/29vcu/?view_only=.

### Results

#### Children’s use of gender as a cue to social status

*Status beliefs as measured by the rope task*. Children placed the boy higher on the rope than the girl, a response pattern in line with societal stereotypes about gender and status. However, this effect was modified by children’s own gender. In the primary model (target gender x participant gender x age), the main effects of target gender, *β* = -1.42, *SE* = 0.31, *t* = -4.62 (95% CI = -2.02, -0.83) and participant gender *β* = -0.72, *SE* = 0.29, *t* = -2.46 (95% Confidence Intervals (CI) = -1.29, -0.15) were subsumed by the interaction of target gender by participant gender, *β* = 1.26, *SE* = 0.41, *t* = 3.05 (95% CI = 0.46, 2.07). Male participants placed the boy higher than the girl, (*M*_boy target_ = 4.57, *SE*_boy target_ = 0.22, *M*_girl target_ = 3.13, *SE*_girl target_ = 0.20, *β* = -1.42, *SE* = 0.29, *t* = -4.86 (95% CI = -1.99, -0.85), a response pattern explicable by either in-group bias or by a belief that gender is a marker of status (or both). Female participants did *not* place the girl higher than the boy (*M*_boy target_ = 3.84, *SE*_boy target_ = 0.22, *M*_girl target_ = 3.67, *SE*_girl target_ = 0.20, 95% CI included zero), suggesting that their responses could not be explained by an in-group bias.

**Fig 2:**
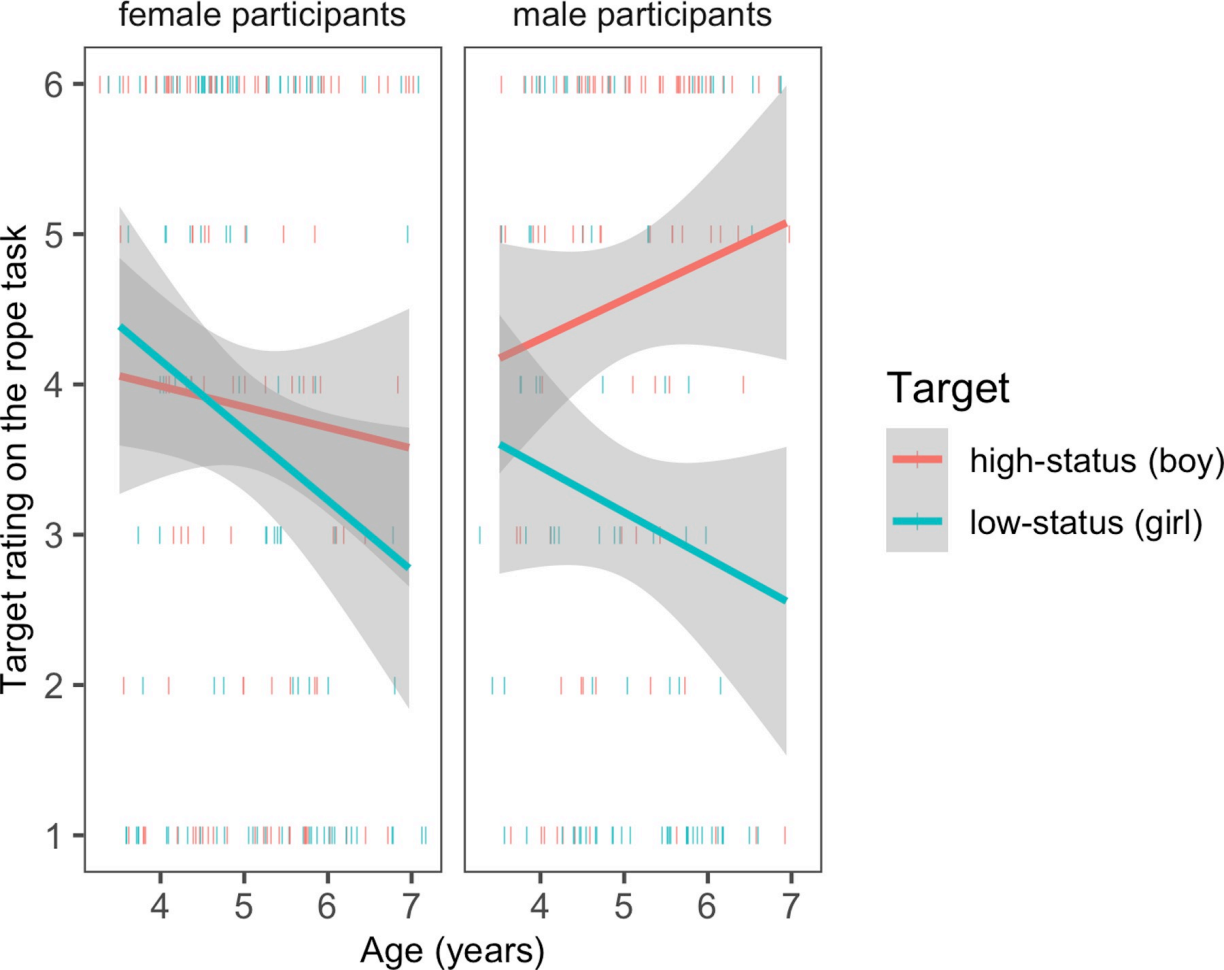
Responses on the rope task as a function of participant gender and age. Shaded areas show 95% confidence bands around the regression lines; dashes represent individual participants.

The 3-way interaction of target gender, participant gender, and age was not significant. However, as illustrated by Fig 2, male participants’ tendency to rate boys as higher status than girls showed some increase with age (although among male participants, slopes for their placement of neither boys nor girls showed significant change across development). In contrast, female participants did not show an own-gender bias at any point in development. In fact, female participants’ ratings of girls showed a significant *decline* across development, *β* = -0.46, *SE* = 0.22, *t* = -2.08 (95% CI = -0.90, -0.03), providing additional evidence that female participants responses could not be explained by in-group bias at any of the ages studied. There was no significant change in female’s ratings of boys across development (95% CI included zero).

*Status beliefs as measured by the wealth-matching task*. Children’s responses on the wealth-matching task differed from their responses on the rope task. On the wealth-matching task, children were *not* more likely to place the boy in the nicer house than to place the girl in the nicer house (*M*_boy target in nicer house_ = 0.49, 95% CI: 0.42, 0.55, comparison to chance responding: *t*(203) = -0.42, *p* = .068). In the primary model (participant gender x age), there was a main effect of participant gender only, *β* = -0.59, *SE* = 0.29, *z* = -1.99, *p* = 0.05. Male participants were more likely to put a boy in the nicer house (*M*_boy target in nicer house_ = 0.58, 95% CI: 0.41, 0.74) than were female participants (*M*_boy target in nicer house_ = 0.44, 95% CI: 0.28, 0.60). This pattern suggests that both male and female responses could be explained by in-group bias. However, as is clear from the Confidence Intervals, placement for neither boy nor girl targets differed from chance, and there were no main or interactive effects with age (95% CIs included zero).

**Fig 3:**
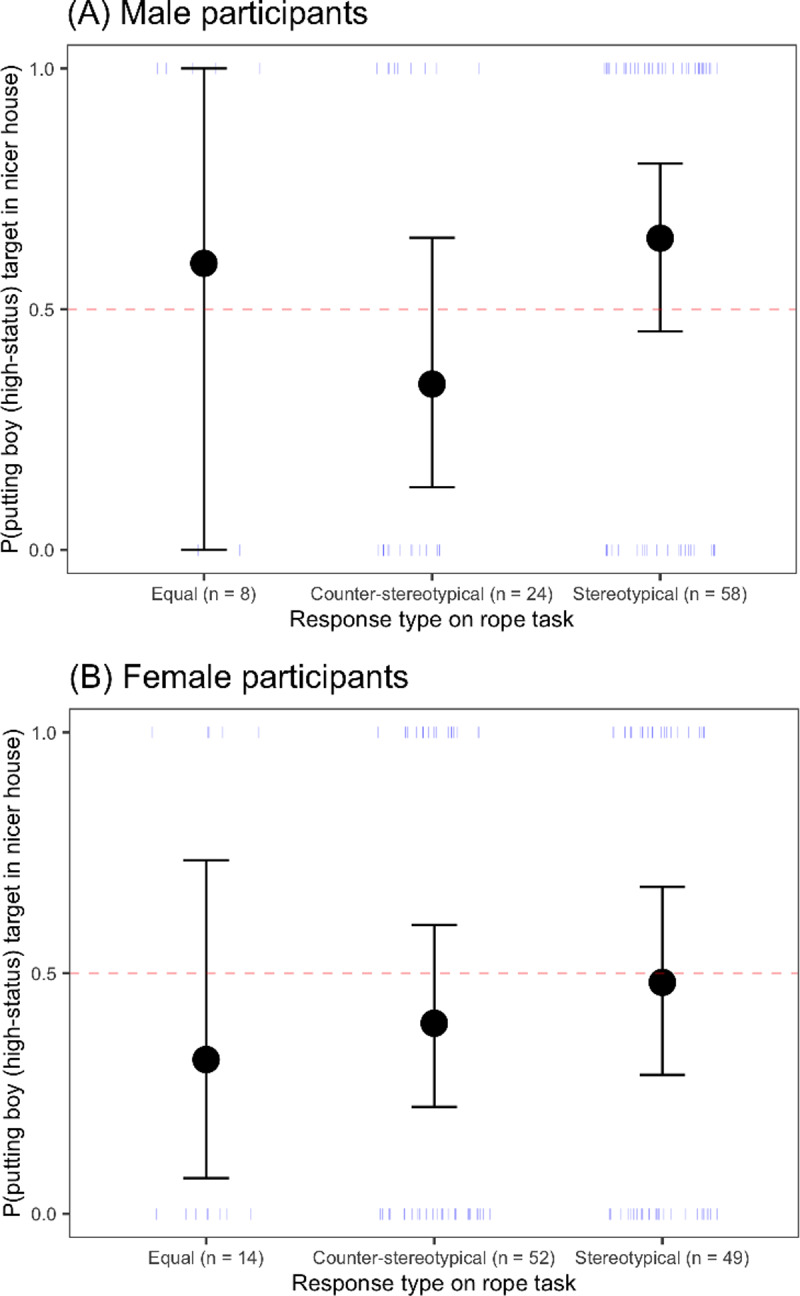
Probability of placing the boy (high-status) child in the nicer house in the wealth-matching task, as a function of response type on the rope task for (A) male participants and (B) female participants. The dotted line represents chance responding. Circles represent the means for each group, and error bars represent 95% confidence intervals around the means; dashes represent individual participants.

*Comparing responses on the rope task and wealth-matching task*. Although the pattern of responses between the two tasks were different, there was evidence of some relation between them (Table 1). In particular, children who placed a boy higher on the rope (a stereotypical response on the rope task) were also more likely to put a boy in the nicer house (a stereotypical response on the wealth-matching task), *β* = 0.75, *SE* = 0.33, *z* = 2.27, *p* = 0.02. However, children did not place boys the nicer house more often than expected by chance, regardless of their response on the rope task (Fig 3).

#### Consequences of children’s use of gender as a cue to social status

**Fig 4:**
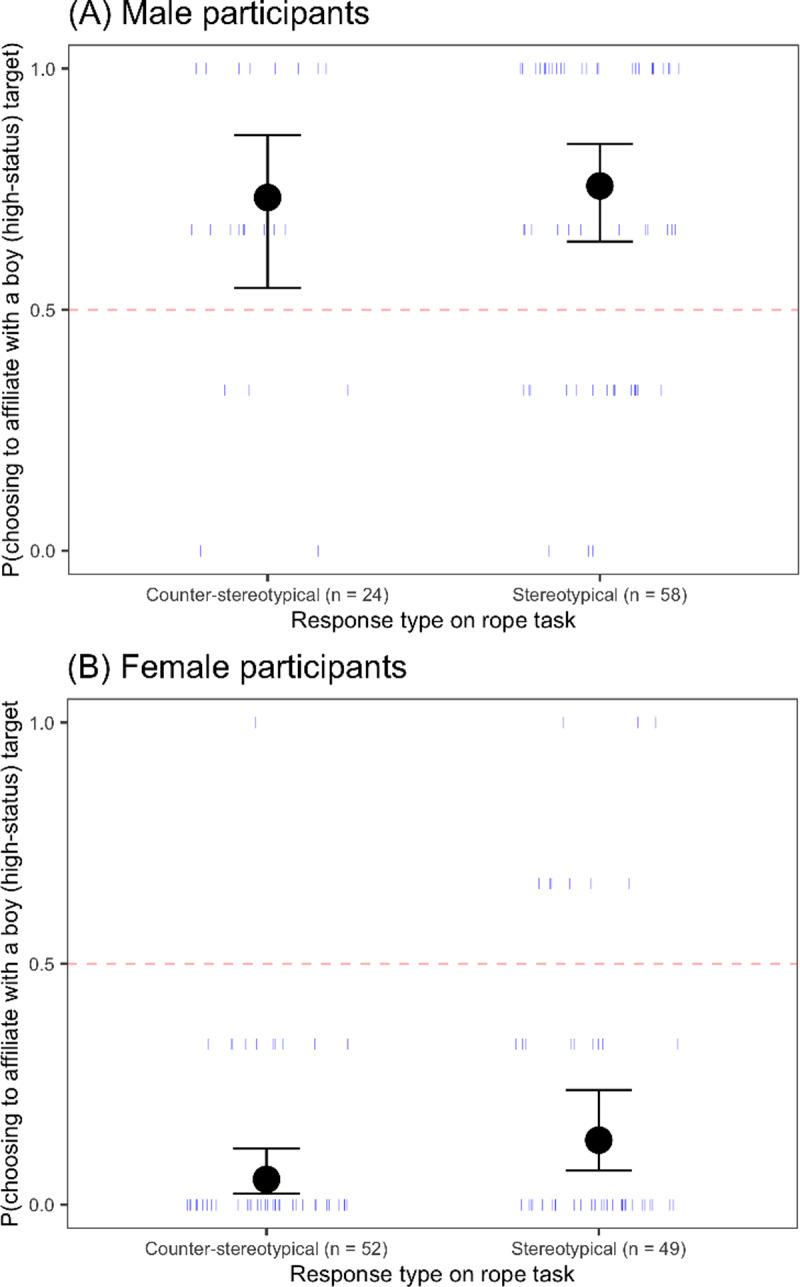
Probability of choosing to affiliate with a boy (high-status) target as a function of response type on the rope task and participant gender for (A) male participants and (B) female participants. Circles represent the means for each group, and error bars represent 95% confidence intervals around the means; dashes represent individual participants.

*Status beliefs as measured by the rope task*. The gender of children’s preferred social partners was unrelated to their responses on the rope task. In the primary model (response on the rope task x participant gender x age), we found only a main effect of participant gender, *β* = -3.89, *SE* = 0.63, *z* = -6.14, *p* < .001. Male participants preferred boys and female participants preferred girls, regardless of participant response on the rope task (Fig 4), a finding fully explicable by in-group bias.

*Status beliefs as measured by the wealth-matching task*. Again, children preferred same-gender social partners regardless of their response on the wealth-matching task. In the primary model (response on the wealth-matching task x participant gender x age), we found only a main effect of participant gender (all other *p*s> .10).

#### Subjective status in children

Participants rated their own status highly; responses for all participants were close to ceiling, and a model including participant gender and age revealed that these ratings did not vary by participant gender (*M*_male_ = 5.5, *SE*_male_ = 0.12, *M*_female_ = 5.3, *SE*_female_ = 0.11), or with age (all 95% CIs include zero). Moreover, subjective status ratings were unaffected by participant’s gendered beliefs about status; when response type on the rope task was added to the above model, there were no significant main or interactive effects (all 95% CIs included zero).

## Study 1 discussion

By assessing children’s status beliefs on the rope task, where social power and wealth were included in the definition of high status, we found that young children used gender to predict the status of others, a novel finding in the literature. Male participants rated boys as higher status than girls across all ages and measures, patterns that can be explained both by status-specific beliefs and by a more general in-group bias (e.g., boys’ belief that boys are better across the board, see [32]).

Even more striking was the result in female participants; although female participants preferred children of their own-gender on the social preference task, they did not rate girls as higher in social status than boys at any age tested. Instead, younger female participants rated boys and girls equally and female participants’ ratings of girls’ status declined significantly with age. Among the oldest ages tested (which was still quite young, as the oldest children were still under 7 years old), female participants rated boys as having more social power than girls (as shown in Fig 2). This pattern is similar to research in other domains, including children’s stereotypes about intelligence [60]. In Bian et al.’s work, 6-year-old male participants rated their own gender as more likely to be “really, really smart,” but 6-year-old female participants were *equally* likely to respond that boys and girls were “really, really smart.” Therefore, male participants’ responses suggested either a general in-group bias or the cultural stereotype that boys are more intelligent (or both). However, female participants’ responses suggested perhaps that they were already struggling to balance their preferences for their own group with social stereotypes that devalue it. Here we found further that before the age of 7, when it comes to social status, cultural beliefs that devalue girls’ status already begin to overwhelm girls’ own-gender preferences in shaping their beliefs about social power.

The role of cultural input is also illustrated by the fact that male and female participants only use gender as a marker of status in the rope task, but not the wealth-matching task. Although we cannot rule out the possibility that a wealth-matching task that used other cues to wealth (e.g., cues that are unlikely to be shared in a heterosexual couple, such as clothing) might be better at detecting children’s beliefs about the covariance of gender and wealth, our results make sense when we consider the types of input that children in our sample were likely to receive. In particular, children in our sample were more likely to see examples of gendered power differentials (e.g., through the media they consume, that is likely to reinforce dominant cultural stereotypes about gender roles) than they are to see examples of gendered wealth differentials (as most of our participants were growing up in heterosexual, two-parent households where wealth was shared by both parents). This explanation is further bolstered by our data illustrating that these same children did use gender to predict status on the rope task, again reinforcing the idea that gender-based status might more firmly be rooted in power, rather than wealth, differentials. This finding also illustrates the utility of the rope task, a modifiable, new measure to assess status beliefs in children that does not rely on wealth-based status alone. It should be noted that in this instantiation of the rope task, where cues to both wealth and social power were presented, we cannot state conclusively that children’s status beliefs about gender are rooted solely in social power differentials. Future research should build upon the work presented here and vary the dimension of status presented (e.g., present *only* wealth or social power or prestige information) to assess exactly which dimension(s) of status children associate most strongly with gender categories.

Although we might not know precisely which dimension(s) of status children relied on when inferring status on the basis of group membership, children’s responses on the rope task indicate that they can use gender to predict status. Yet we did not find a relation between their gendered status beliefs and gender preferences. Rather, children’s gender preferences were strongly driven by in-group biases and were unaffected by children’s use of gender as a cue to status. This is important, as it sheds light on the processes by which social status influences preference and bias. In particular, this finding illustrates that viewing certain social groups as higher status does not mean that those groups will inevitably be preferred, and perhaps that status beliefs are necessary for the development of bias in some, but not all, social domains (a supposition we explore more in Study 2). Finally, children’s assessment of their own status was relatively immune to the effects of their gendered beliefs about status, with both male and female participants expressing positive evaluations of their own status.

## Study 2

Understanding the development of preferences and biases that disadvantage groups stereotypically thought of as lower in status is particularly relevant when thinking about racial identity and racial inequality. In fact, much of the previous work on children’s real-world status concepts has examined whether children use race to predict the status of others (e.g., [27,28]). However, much of this work has focused on the use of race as a cue to wealth in particular, and has not examined whether race might be viewed as predictive of other dimensions of status as well (but see [22] for work on the conflation of race and prestige in children). Moreover, viewing Black individuals as lower status has been hypothesized as foundational for the development of pro-White/anti-Black biases [19]. Although children’s pro-wealth and pro-White attitudes seem to be related (at least when implicit pro-White attitudes are considered: [42,43]), there is limited evidence testing the direct relation between status *beliefs* about race and pro-White preferences. The single empirical study on this topic, conducted in South Africa, found only a weak relation between status beliefs and racial preferences [27]. Therefore, in Study 2, we aimed to clarify whether children use race to predict status, what status dimensions were most important to this relation, and how these race-based status beliefs affected both racial preferences as well as subjective status beliefs.

### Method

#### Participants and procedures

Methods for recruitment and sample size determination were identical to Study 1 (see SOM for further information). Similar to Study 1, participants in this sample were aged 3.5–6.9 (*M*age = 5.01; *N* = 205; 56.1% female; White: 34.6%; Hispanic: 10.7%; Asian: 12.2%; Black or African-American: 12.7%; Multiracial: 14.6%; other: 2.4%; not provided: 12.7%). Child race was determined by parental report, and parents reported similar employment and educational background to Study 1. Sample characteristics did not vary between participants who were identified by parents as White, or as a member of one or more racial-ethnic minority groups (comprising Black/African-American, Asian, Hispanic, Multiracial, and other). The pre-registration, protocols, and data, and analytic codes are available on OSF using the links given in Study 1.

All methods were identical to Study 1, except instead of presenting a gender contrast as in Study 1, the stimuli presented a Black-White racial contrast. Children in the CAFE stimulus set had been identified by their parents as European-American or as African-American, and all stimuli were gender-matched to the participant gender (i.e., female participants saw White and Black female targets, male participants saw White and Black male targets). Again, all stimuli were closed-mouth neutral-faced images taken from the CAFE set [55,56], except for the social preferences task, in which images were taken from a Google search. For authorized Databrary users, stimuli can be viewed at: https://nyu.databrary.org/volume/599. At no point during the task were any of the stimuli labeled by racial group membership, enabling us to assess whether children attended to race spontaneously.

#### Analysis plan

Data were analyzed following the same general analysis plan as in Study 1. Again, for the rope task, participants who failed to pass the comprehension check for either the high-status or low-status comprehension cards were excluded from analyses (excluded *n* = 10; final *N* = 205). However, because our participants’ racial identities (White or racial-ethnic minority) did not exactly match the racial identities of the target stimuli we used (White and Black children), we ran two models (as planned in our pre-registered analyses). Our first model did not include participant racial-ethnic identity, but our second model did.

In the United States, the White racial majority frequently holds more resources and power than racial-ethnic minority groups [17,18], thus target race and participant racial-ethnic background were treated as proxies for social status where White targets and White participants were considered to be members of the “high-status” racial group and Black targets and racial-ethnic minority participants were considered to be members of the “low-status” racial group. As a note, the cleanest test of our hypotheses would be to restrict participants in Study 2 solely to White and Black/African-American participants (in order to make it more directly comparable to the in-group/out-group distinction possible in Study 1 where we examined male and female children’s use of gender as a cue to social status). However, due to insufficient sample size, and the practical and ethical difficulties with recruiting only White and Black/African-American participants, the analyses reported in the main text used the racial-ethnic categories of White and racial-ethnic minority participants only, making the sample one of convenience in this respect. Although not ideal, using this racial-ethnic categorization still allows for a test of whether preferences for the high-status group reflect in-group biases only (in which case, we might find status-related beliefs and preferences only among White children, for whom the high-status group is also the in-group) or more generalized beliefs related to status (in which case, we might find similar patterns among racial-ethnic minority children, for whom the high-status group is *not* the in-group). Whenever possible we ran additional analyses that were restricted to White and Black/African-American participants; given the small sample size for these analyses they should be interpreted with caution, and therefore we report the results of these restricted analyses in the SOM only.

### Results

#### Children’s use of race as a cue to social status

**Fig 5:**
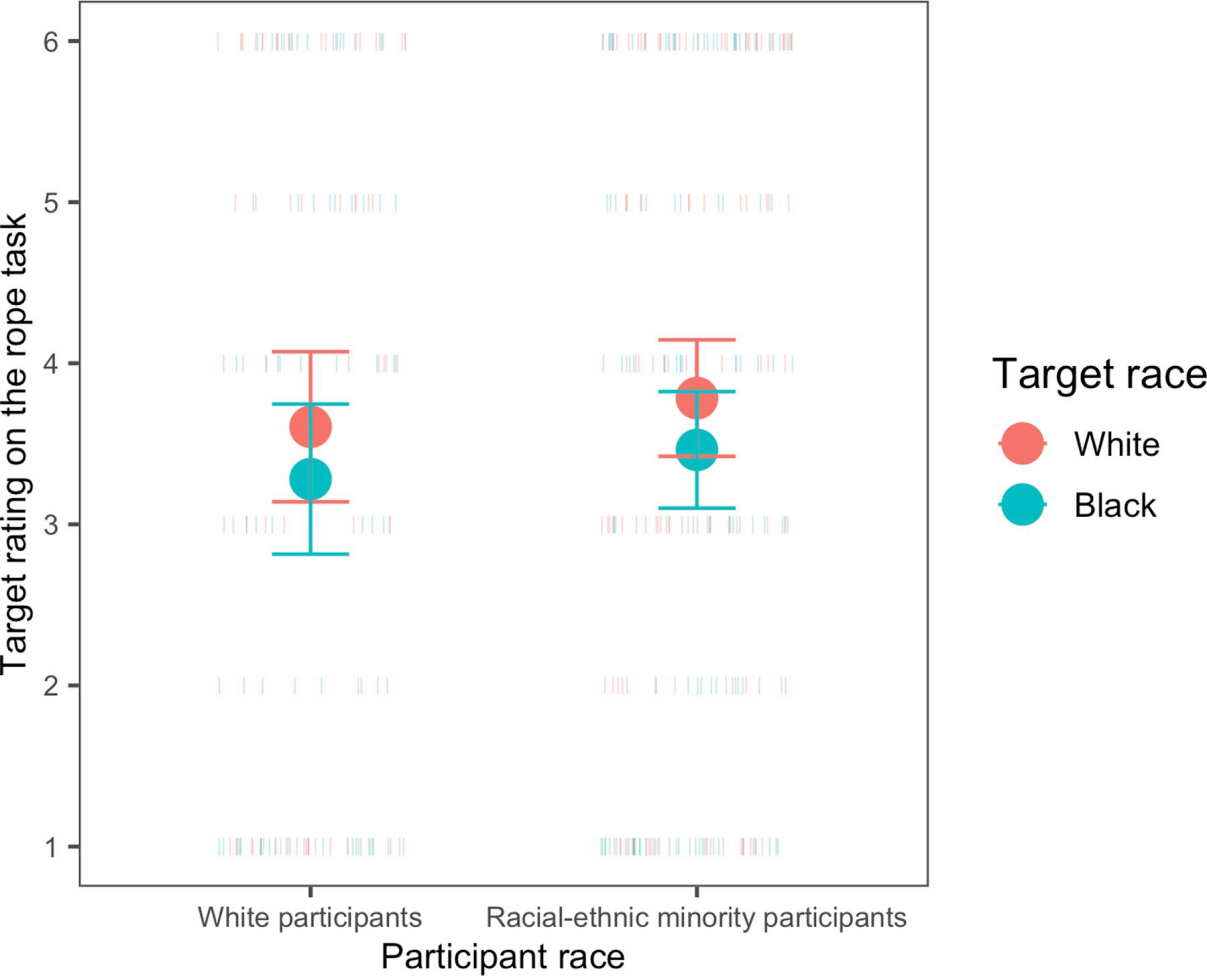
Responses as a function of participant race on the rope task. Circles represent the means for each group, and error bars represent 95% confidence intervals around the means; dashes represent individual participants.

*Status beliefs as measured by the rope task*. Children did *not* use race as a cue to social status on the rope task. In the first model (target race x age), there were no main or interactive effects of target race (both 95% CIs included zero). Ratings of both White and Black targets decreased with age (main effect of age, *β* = -0.36, *SE* = 0.16, *t* = -2.28 (95% CI = -0.66, -0.05)). When participant race was added to create the secondary model (target race x age x participant race), we found no main or interactive effects (all 95% CIs include zero: Fig 5).

*Status beliefs as measured by the wealth-matching task*. Children’s responses on the wealth-matching task differed from their responses on the rope task. On the wealth-matching task, children *did* use race as a cue to status; they were more likely to place the White child in the nicer house than to place the Black child in the nicer house (*M*_White target in nicer house_ = 0.68, SE _White target in nicer house_ = 0.04; 95% CI: 0.62, 0.75, comparison to chance responding: *t*(213) = 5.71, *p* < .001). In the primary model (participant race x age), there were no main or interactive effects of participant race or age on their likelihood of saying that the White child lived in the nicer house (all 95% CIs include zero, and both White and racial-ethnic minority participants responded that the White child lived in the nicer house more often than expected by chance). Although White participants’ responses could be explained by either in-group bias or by a belief that race is a cue to status (or both), racial-ethnic minority participants’ responses cannot be explained by in-group bias and thus are more likely to indicate the belief that race is a marker of status.

**Fig 6:**
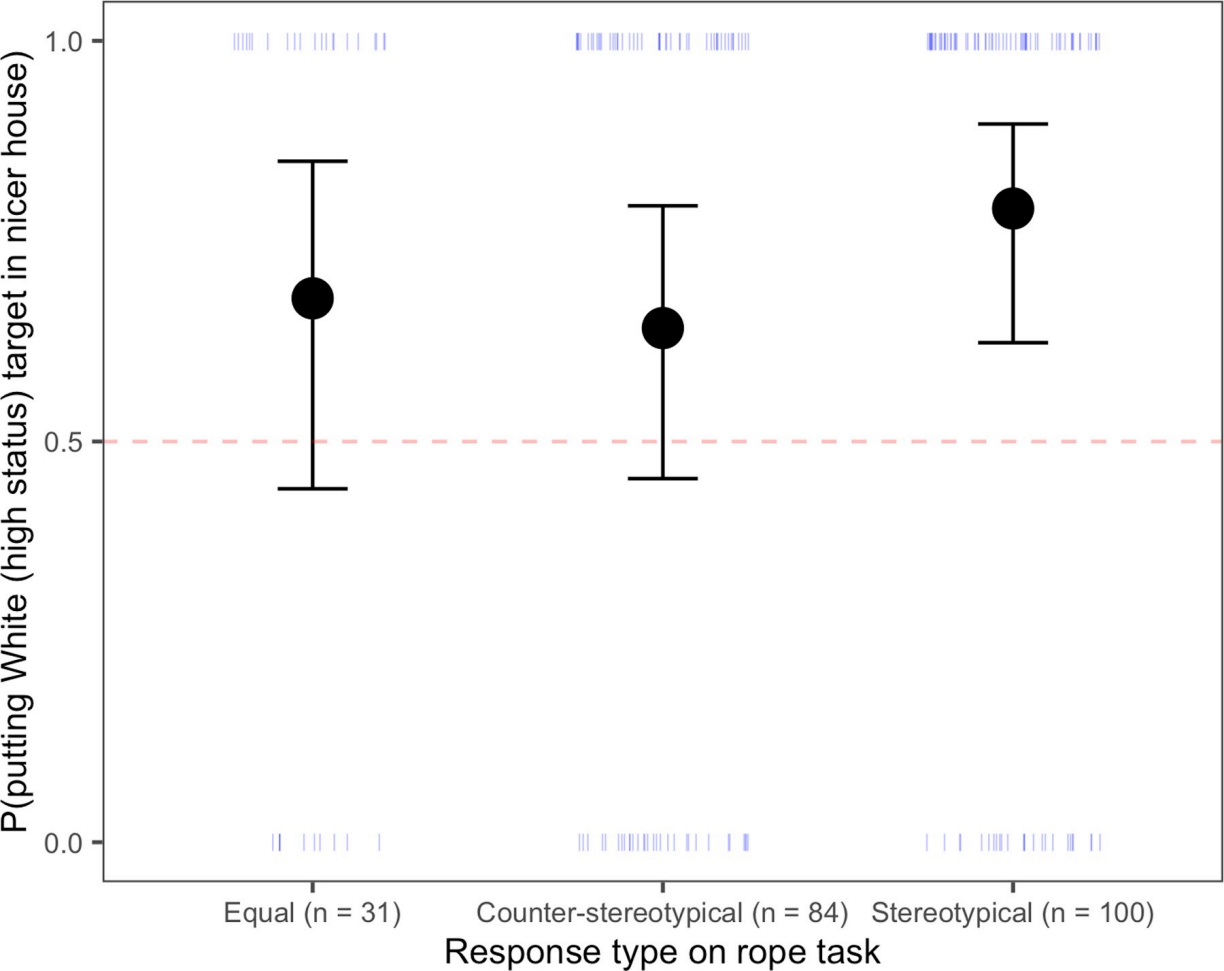
Probability of placing the White (high-status) child in the nicer house in the wealth-matching task, as a function of response type on the rope task. The dotted line represents chance responding. Circles represent the means for each group, and error bars represent 95% confidence intervals around the means; dashes represent individual participants.

*Comparing responses on the rope task and wealth-matching task*. Although the pattern of responses between the two tasks were different, there was evidence of some relation between them (Table 2). Children who placed a White child higher on the rope task (a stereotypical response on the rope task) were also more likely to put the White child in the nicer house (a stereotypical response on the wealth-matching task), *β* = 0.71, *SE* = 0.37, *z* = 1.93, *p* = 0.05. Only participants who responded in this stereotypical manner on the rope task were above chance in placing the White child in the nicer house on the wealth-matching task (Fig 6). Finally, responses on the two status tasks showed greater convergence with age, *β* = 1.3, *SE* = 0.43, *z* = 3.0, *p* < 0.001 (see Fig SOM3 in the S1 File).

**Table 2:** Number of Study 2 participants who provided a given response type for the rope task and wealth-matching task, separately for White and racial-ethnic minority participants. Table includes only participants whose parents reported child race (*n* = 186).

	*Response type on Rope task*		
	*Equal*	*Stereotypical*	*Counter-stereotypical*
**Rope task**	White & Black child in same position	White child in higher position than Black child	Black child in higher position than White child
Racial-ethnic minority participants	*n* = 22	*n* = 49	*n* = 45
White participants	*n* = 8	*n* = 35	*n* = 27
**Wealth-matching task**	n/a	White child in nicer house	Black child in nicer house
Racial-ethnic minority participants		*n* = 86	*n* = 36
White participants		*n* = 47	*n* = 24

#### Consequences of children’s use of race as a cue to social status

*Status beliefs as measured by the rope task*. Children’s responses on the rope task and age predicted their choices of social partners. In an analysis examining the main and interactive effects of participant response on the rope task and age, children became more likely to choose a White social partner with age (main effect of age, *β* = 0.44, *SE* = 0.2, *z* = 2.19, *p* = 0.03). Participants who responded in a stereotypical manner on the rope task (as compared to those who responded in a counter-stereotypical manner) were also more likely to choose a White social partner (main effect of the response participants gave on the rope task, *β* = 0.7, *SE* = 0.26, *z* = 2.66, *p* = 0.01).

**Fig 7:**
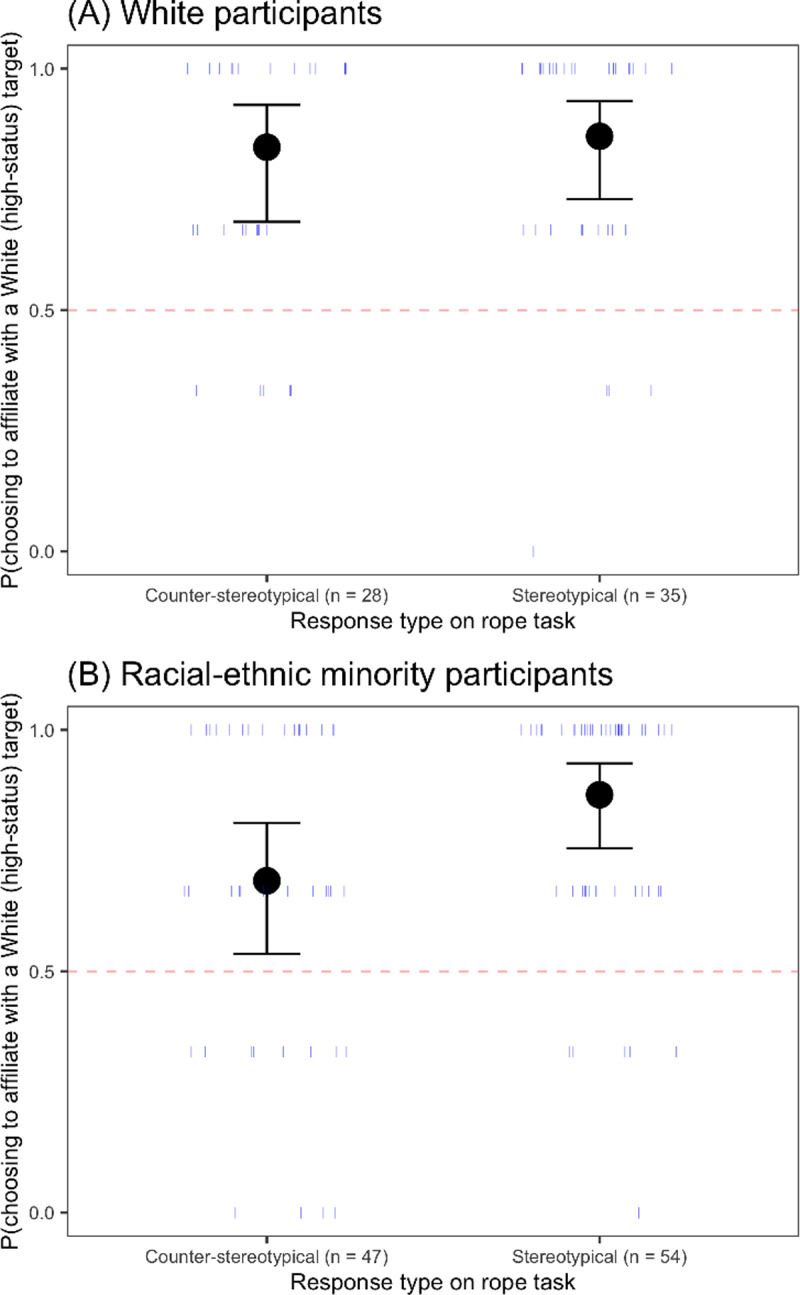
Probability of choosing to affiliate with a White (high-status) child as a function of response type on the rope task and participant race for (A) White participants and (B) Racial-ethnic minority participants. Circles represent the means for each group, and error bars represent 95% confidence intervals around the means; dashes represent individual participants.

When participant race was added to create the secondary model (response type on the rope task x age x participant race), we found the same main effect of age as before, *β* = 1.3, *SE* = 0.48, *z* = 2.7, *p* = 0.01. We also found marginal effects of participant race, *β* = -0.78, *SE* = 0.44, *z* = -1.78, *p* = 0.07, and a two-way interaction of age by participant race, *β* = -1.04, *SE* = 0.54, *z* = -1.91, *p* = 0.06, which were both subsumed under a marginal three-way interaction of age, participant race, and response type on the rope task, *β* = 1.38, *SE* = 0.76, *z* = 1.82, *p* = 0.07. Regardless of response type on the rope task, White participants were above chance in selecting a White affiliation partner (Fig 7A), a response pattern best explained by in-group bias. Although the pattern was similar in racial-ethnic minority participants, those who responded in a stereotypical manner were slightly more likely to select a White affiliation partner than those who did not (Fig 7B). This is consistent with the possibility that partner preferences among racial-ethnic minority participants were affected by the belief that race is a marker of status, although these findings were statistically weak (similar to [27]) and should be interpreted with caution.

*Status beliefs as measured by the wealth-matching task*. In contrast, status beliefs as assessed by the wealth-matching task did not predict social preferences, either on their own or through interaction with any other variables (*p*s > .10 for all main and interactive effects including response type on the wealth-matching task). As before, only age was related to an increase in selecting a White social partner.

#### Subjective status in children

Participants rated their own status highly; responses for all participants were close to ceiling, and a model including participant race and age revealed that these ratings did not vary by participant race (*M*_White_ = 5.33, *SE*_White_ = 0.14, *M*_Racial-ethnic minority_ = 5.26, *SE*_Racial-ethnic minority_ = 0.11) or with age (all 95% CIs include zero). Subjective status ratings were affected by participant’s beliefs about race and status, but in a somewhat counter-intuitive manner. When response type on the rope task was added to the above model, participants who responded in a counter-stereotypical manner rated themselves as higher in status than those who responded in a stereotypical manner (main effect of response type, *β* = -0.61, *SE* = 0.29, *t* = -2.07 (95% CI = -1.17, -0.04)). An interaction of participant race and response type, *β* = 0.74, *SE* = 0.38, *t* = 1.95 (95% CI = 0.01, 1.46) showed that White participants who responded in a stereotypical fashion on the rope task rated their own status as lower than White participants who responded in a counter-stereotypical fashion, a pattern we discuss further in the Study 2 Discussion.

## Study 2 discussion

In Study 2, we found a substantially different pattern of responding to Study 1. In Study 1, children used gender to predict status when status included information about social power (on the rope task) but not when it was defined solely in terms of wealth (on the wealth-matching task). In contrast, children in Study 2 did not use race to predict status when status included information about social power (on the rope task) but did when status was defined solely in terms of wealth (on the wealth-matching task). By demonstrating that children used race to predict social status, at least when status was represented by wealth, Study 2 replicated previous work [26,27,28].

Why did children use race to predict status on the wealth-matching task, but not on the rope task? Although there are several plausible reasons for the discrepant results across these two tasks, we believe it is most likely due to differences in how the tasks were constructed. As a forced choice task that is set up to appear zero-sum (e.g., there are two target children and two target houses), the wealth-matching task might have pushed children away from engaging in more egalitarian responding, such as saying that both the White and the Black child lived in the same house. Instead, this task might have led children to fall back on existing cultural and stereotype knowledge to make their decision, even if that knowledge was not strongly held. In contrast, by giving children the opportunity to respond in a more egalitarian manner, the rope task might have been less able to detect subtle, just emerging stereotype knowledge, and instead encouraged children to respond in line with how they think things *should* be (i.e., fair). Additionally, it is possible that race was not made salient enough in the rope task for children to use race to draw inferences about the relative status of the Black and White targets. Children at these young ages often do not spontaneously attend to or remember race [61]. Thus, race might only have been salient enough to engender race-based inferences about status when children were presented with a forced-choice option between two side-by-side child stimuli that varied in race and two side-by-side house stimuli that varied in quality, as in the wealth-matching task.

Even though children did not appear to use of race as a marker of status on the rope task, individual variation on the rope task was indeed (weakly) related to pro-White bias (whereas variation in the wealth-matching task did not predict pro-White bias). This raises the additional possibility that perhaps only stronger forms of racialized beliefs (i.e., those detectable on the rope task) relate to affective bias, and that this bias is not solely explicable by a preference for historically wealthy, resource-rich groups. Although this result is only a first step, understanding how variation in race-related status beliefs relate to the development of racial bias is important in understanding exactly when and why members of lower-status groups show out-group preferences or dislike of their in-group.

We found one unexpected result related to subjective status; White participants who used race as a cue to status rated their own status lower than White participants who did not use race in this way. It is unclear whether children who did use race as a cue to status have more realistic expectations about what status means, or whether they are directly comparing themselves to other White people, whom they perhaps consider to be high status. Although we were unable to collect detailed data on participant’s socio-economic status here, including this information in future research is essential to understand the development of children’s subjective status beliefs (as in 47,48]).

## General discussion

The present studies examined the development of children’s use of gender and race as cues to social status, and how use of these cues relate to both children’s inter-group attitudes and beliefs about their own place in the social milieu. Children used both gender and race as cues to social status, but they did so on different tasks. Moreover, whereas use of gender to predict status did not relate to gendered social preferences, viewing race as a cue to status was weakly related to racial preferences, at least among racial-ethnic minority children. Finally, although children held group-based beliefs about status, they did not apply these beliefs to themselves, suggesting a disconnect between children’s abstract beliefs about the structure of their world and their beliefs about their own identities.

### The development of group-based beliefs about status

Although children as young as 3.5-years-old used both gender and race to predict social status, they used these cues differently on different tasks. Children used race to predict who lived in a fancier house (wealth-matching task), and responses on this task were stable across age in our sample of 3.5- and 6.9-year-old children. This pattern replicates previous findings from both the U.S. [26,28] and South Africa [27], and is the first to find evidence of this tendency in children as young as 3.5-years-old.

Consistent with previous research, children did *not* use gender to predict who lived in a nicer house on the wealth-matching task. However, using the rope task, a new measure of status beliefs where status was defined in terms of both resources and social power, children did indeed use gender as a cue to status. Overall, children assigned boys higher status positions than girls on this task, and this tendency increased with age. This age-related change was driven by a decrease in participants’ views of girls’ social status, a pattern found in both male and female participants, but most strongly in female participants. Female participants’ tendency to assign lower social status to girls with age is striking, as this pattern runs counter to their own in-group biases. Indeed, not only did female participants show a systematic decrease in their assessment of other girls’ status across age, they never showed an own-gender advantage, even at the youngest ages tested (although they did show gender in-group biases on other measures). These patterns indicate that girls begin to view boys as holding greater social power from quite early in development.

Future research should examine how these status beliefs about gender develop so early in childhood. One possibility is that children’s early-emerging tendency to use physical size as a cue to dominance [7,8] combines with the fact that men, on average, are physically larger than women, to scaffold the belief that men are socially dominant to women. Children as young as 3- and 4-years old draw connections between physical strength and social dominance [31], making it plausible that they map these intuitions onto gender categories as well. Especially in conjunction with children’s media, where girls and women are often depicted as helpless, submissive, and weak [33,34,35], these intuitions could contribute to our finding that children view boys as more socially dominant than girls.

Finally, our goal across these two studies was to examine children’s status beliefs about gender and race separately (e.g., choosing Latinx stimuli for the gender condition, as children’s beliefs about status for Latinx social categories appear later in development: [62]). However, in the real-world there are important intersectional processes that affect the content of individual’s beliefs and attitudes [62,63]. For example, children as young as 5-years-old are less likely to view Black women (as compared to White women) as feminine [63]. Determining whether the content of children’s status beliefs are affected in a similarly intersectional process will be an important area for subsequent research.

### The consequences of status beliefs

On the preferences task, children showed strong own-gender preferences, whether or not they used gender as a cue to social status. Racial-ethnic minority children who used race to predict social status expressed slightly stronger pro-White bias than did those who did not use race in this way. In contrast, White children showed pro-White bias regardless of their status beliefs, likely because, among this group, bias could stem from general in-group bias as well as from status-related beliefs. This is the first evidence to link race-based beliefs about status to pro-White preferences in the United States; however, it is worth noting that this link was fairly weak and found among only some populations of children.

In the present study, among Black children in particular, there was no relation between the tendency to use race as a cue to social status and pro-White bias (presented in detail in the SOM). Overall, Black participants did not choose to affiliate with a Black partner more often than expected by chance (suggesting, in line with previous work, a weaker in-group bias than is present in White children). Although preliminary given the small number of Black participants in this study, the lack of relation between Black children’s in-group bias and their status beliefs is intriguing, as theorists have suggested that Black individuals sometimes hold weaker in-group biases because they accept (rather than reject) hierarchies that systematically disadvantage them (e.g., [19,50]). Previous work has found a much stronger relation between Black children’s pro-wealth attitudes and their implicit pro-White biases [42,43]. Here though, when we examine children’s status beliefs (rather than status attitudes) we found no relation between status beliefs and racial preferences among Black children, and overall, relatively little evidence for a direct relation between status beliefs and social preferences more generally, across populations and across tasks (findings that are in line with previous research:[27]).

One possible explanation for the relatively weak relation between status beliefs and bias is that status beliefs about real-world social groups are necessary but insufficient to engender attitudes and behaviors that disadvantage stereotypically lower-status groups. For example, children who observe that race and wealth often covary in the world around them might come to believe that White people are more likely to be rich and Black people more likely to be poor either because of some intrinsic and unchangeable factor inherent to racial minorities, or because of structural or historic factors that disadvantage minority groups (including racist policies, such as segregation, redlining, and discrimination). In this way, status beliefs might only lead to systematic devaluation of minority groups and pro-White bias in the former case, when explanations for why race and status covary focus on inherent factors rather than structural ones (for discussion of children’s tendency to rely on inherent explanations, see [64]). Overall, our data illustrate the need to better understand what additional processes (e.g., children’s beliefs about the causal mechanisms underlying status hierarchies, children’s tendency to appeal to inherent—rather than structural—explanations, etc.) might mediate the relations between representations of hierarchies and their social consequences (for detailed discussion on this topic, see [65]). Understanding the precise processes by which status beliefs affect social bias (if and when they do) is important for the development of future interventions that aim to reduce prejudice and bias by targeting malleable aspects of cognition (such as the types of explanations given for societal stereotypes). Finally, it would be useful to examine pro-White and anti-Black bias separately (as they were confounded in the present measure of social preference) to clarify how status beliefs relate to preference and bias across diverse populations of children. More precisely untangling the relations between in-group biases, status beliefs, and racial attitudes across development is of crucial importance, both to enhance our understanding of how these various inter-group phenomena unfold and to develop ways of utilizing this knowledge to reduce the development of stereotypes and prejudices.

### Subjective status in children

Children in this sample had positive views of their own status. This finding aligns with previous research on the knowledge-behavior disconnect (e.g., [66]) suggesting that, especially when it comes to self-evaluation, preschool-aged children are quite positive (e.g., [49]), at least relative to older children. Recent cross-cultural research investigated 4- to 18-year-olds across four countries (Argentina, Ecuador, India, and the United States) and found that children’s assessments of their subjective status decreased with age [67], suggesting that we might see a similar decrease in self-evaluation should we test older children on the rope task. However, because we did not collect detailed data on an objective measure of children’s status (e.g., family SES as assessed via income) and because our participants were primarily from highly educated and fully employed families, we do not know whether children’s subjective status assessments are related to their objective SES, or whether young children’s subjective assessments might vary more widely across more economically diverse populations of children (as has been observed in economically diverse samples of older children: [47,48,68]).

Additionally, although very young children might be buffered from negative subjective status beliefs about themselves, they do seem aware of the relative wealth-based status of their families or communities. For instance, when asked how many resources their family has, children’s responses correlated with the median income in their community [48], suggesting that children might be able to provide a more accurate, and less rosy, evaluation of their relative status when asked to make evaluations of a more distal aspect of themselves (e.g., their family or community, instead of themselves). Both of these limitations should be addressed in future research.

Although we were unable to collect detailed socio-economic information about the children in our sample, we were able to collect information about gender and racial identity. Children’s responses on the rope task did not relate to their views of their own status among children from groups that are stereotyped as lower status (i.e., female and racial-ethnic minority children). This suggests that, at least in early childhood, children are buffered from perceptions of status that might harm them (although we do not suggest that children are similarly buffered from disadvantage or barriers imposed by actual structural inequality in the world around them). However, by adulthood, individuals are willing and able to make negative evaluations of their own status, and subjective social status becomes one of the best predictors of health, well-being, and even mortality risk [46,54], over and above objective status measures such as SES. More research is needed to understand how subjective social status comes to have such profound effects across development, and exactly when children become willing or able to make negative evaluations of their own status. Moreover, although the links between subjective status and health outcomes are certainly compelling, there are a variety of ways that status might affect other outcomes as well, such as children’s beliefs about what educational or occupational opportunities are realistic options for them [e.g., 22, 23]. By better understanding when children start to apply group-based status hierarchies to themselves, and by understanding how that developmental process unfolds, we can foster healthy identity development among children from vulnerable, often stigmatized groups.

As we demonstrated here, the processes that give rise to a variety of social and individual consequences originate in early childhood—young children reason systematically about social status and map it on to those around them, with implications for inter-group attitudes and subjective status beliefs. This study provides clear evidence that children are aware of societal stereotypes about status across multiple social domains, replicating and extending previous work, yet many important questions remain. For instance, although our data across tasks suggest that children link gender to status hierarchies based in social power, rather than in wealth, additional research is necessary to better understand the relative contribution of these different dimensions of status, both for gender and race, as well as for other social domains. One of the strengths of the rope task is its adaptability to investigate these types of questions; experimenters can provide participants with different dimensions of status (e.g., physical size or prowess, access to resources, wealth-holding, social or decision-making power, prestige or social influence, etc.) and stimuli that vary across a variety of social domains (e.g., race, gender, ethnicity, religion, immigration status, etc.). Although this study was a first step, and thus we chose to include two dimensions of status to increase the chances that young children would attend to status information, future work should systematically vary both the status dimension presented as well as the social domain in question. In this way, we can start to tease apart the particular status cues that children use to make inferences about the status of others across different social domains, as well as the developmental progression of these associations across development. Future research can build on our findings to develop a fuller picture of the development and consequences of status beliefs, and in particular, to understand how children come to apply status beliefs not only to the world around them, but also to themselves.

## Supporting information

S1 File (DOCX)

S1 Data (DOCX)
