# Supplementary material for: Children's use of race and gender as cues to social status
Source: PLoS One. 2020 Jun 22;15(6):e0234398. doi: 10.1371/journal.pone.0234398 (PMC7307787; doi:10.1371/journal.pone.0234398)
Supplement: S1 File — (DOCX) [file pone.0234398.s001.docx]

**Supplemental Online Materials**

Here we report details of a measure administered but not described in the main text, all deviations from the pre-registration in full details, and additional analyses that were secondary to the primary goals of the study. These include: (1) results of the wealth-liking task across Studies 1 and 2, (2) effect of trial number on the wealth-matching task across Studies 1 and 2, (3) age-related changes on concordance between the two status tasks in Studies 1 and 2, and (4) analyses focused on Black and White participants in Study 2. None of the results presented here change the interpretations offered in the main text.

**Method**

**Details of wealth-liking task (administered, but not included in the main text)**

After participants completed the wealth-matching task, they completed two trials of a wealth preference task to assess whether they preferred targets associated with high wealth items. In a trial, participants were presented with two houses; one of the houses appeared well-maintained and the other house appeared more run-down. Participants were then presented with a pair of targets that were matched in race and gender, and were told that one of the kids lived in one of the houses and the other kid lived in the other house. Participants were then asked which kid they liked. In Study 1, participants saw a pair of Hispanic boys and a pair of Hispanic girls on separate trials. In Study 2, participants saw a pair of White targets and a pair of Black targets, gender matched to the participant’s gender, on separate trials. Across both studies, which pair of targets was presented first was counterbalanced between participants.

**Deviations from the pre-registered analysis plan.** We completed the analyses as indicated in the pre-registration, with four exceptions. First, we originally planned to analyze data for Study 1 and Study 2 together (treating gender and race as a between-subjects condition variable). However, we predicted different patterns of results across our status and social preference tasks for gender versus race, and realized in retrospect that the results were clearer and more digestible by separating presentation of the gender and race results. Second, and as related to the first point, in Study 1 (when examining status beliefs and social preferences about gender) we chose only to include a model where participant gender was included. Because gender is a salient and meaningful category to young children (e.g., [20]), and because we used stimuli that matched participants’ own gender identity (e.g., stimuli were of male and female children and participant gender identity was male or female), analyses that take gender in-group biases into account (by including participant gender) were most appropriate. In Study 2, where our participant racial identities (White or racial-ethnic minority) did not exactly match our stimuli (White and Black children), we stuck to the pre-registered analyses and ran two models, a first that did not include participant racial-ethnic identity, and a second one that did. Third, instead of testing age as a main effect, we included it as an interactive effect to better examine developmental changes across our sample. Fourth, we included study number as a random effect in all models. We made this last deviation from the planned analyses because we chose to analyze data for all participants within Study 1 together, but data were actually collected across two versions of the study (1a and 1b). The a and b versions were identical apart from the inclusion of one additional task in Study 1a, presented directly after the rope task was completed, that aimed to assess children’s essentialist beliefs about gender (or race, in Study 2). Due to problems with this task (many children failing warm-up training trials), we opted not to consider this variable further in this set of studies. As our primary measure of interest in this study was the rope task, which preceded this essentialism task and thus could not be affected by the inclusion of this additional task, we opted to combine data from Studies 1a and 1b to increase sample size and our power, allowing us to better examine the interactive effects of participant characteristics (i.e., participant membership in high or low status groups). However, to be conservative and adjust for any possible effects of this additional task on tasks that came after it, we included study number as a random effect in all models (as a note, we also found no differences between Study 1a and 1b in the wealth-matching task or social preference task responses, both of which followed the essentialism task when it was administered).

**Results**

**Wealth-liking task**

In Study 1, there was no effect of presentation order (either as a main effect, or as an interaction with target status); therefore, data were collapsed across presentation orders. In contrast to previous research [28], participants in Study 1 exhibited no preference for the target associated with the well-maintained (high-status) house over the run-down (low-status) house (all *p*s > .10). In Study 2, there was an interaction of presentation order and target status, $\beta$ = -0.84, *SE* = 0.39, *z* = -2.14, *p* = .03. Among children who were first asked about a pair of White targets, and then asked about a pair of Black targets, there was no preference for the target presented with the nicer house for either pair. In contrast, among children who were first asked about a pair of Black targets, and then asked about a pair of White targets, participants were above chance in selecting the target presented with the nicer house, but only for White targets (*M* = 0.64, *SE* = 0.05). These children showed no preference for the Black target paired with the nicer house (*M* = 0.55, *SE* = 0.05). Thus, across Studies 1 and 2, we find inconclusive evidence that children strongly prefer wealthier others.

**Wealth-matching task: Role of trial number**

As described fully in the main text, participants completed two trials of the wealth-matching task, where the only thing that varied between trial one and trial two was the order in which the target child cards were presented below the houses (i.e., in trial one, the high-status target was on the left and the low-status target was on the right, and vice versa for trial two). We found order effects on this task that made it difficult to interpret responses to the second trial; therefore, we reported results for the first trial only in the main text.

In Study 1, when we examined both trials in a model that included as fixed factors the main and interactive effects of trial number and age, we found main effects of trial number, $\beta$ = -0.61, *SE* = 0.21, *z* = -2.88, *p* = .004 (Fig SOM1), and an interaction of trial number by age, $\beta$ = 0.72, *SE* = 0.24, *z* = 3.07, *p* =.002 (Fig SOM2). Participants were at chance on trial one (as described in the main text) and were more likely to say that the female - lower status - target lived in the nicer house on trial two. With age, the tendency to put the female target in the nicer house decreased and by approximately 5-years-old children were at chance on trial two as well. It is possible that we found this age-related change because older—as compared to younger—children might have held both stronger beliefs that status and gender covary, as well as less compulsion to respond in a “fair” manner (i.e., after putting the high-status target in the nicer house on trial one, subsequently putting the low status target in the nicer house on trial two).

In Study 2, when we examined both trials in a model that included as fixed factors the main and interactive effects of trial number and age, we only found a main effects of trial number, $\beta$ = -0.45, *SE* = 0.21, *z* = -2.17, *p* = .03 (Fig SOM1, SOM2). Participants were above chance on trial one in saying the White target lived in the nicer house (as described in the main text) and were at chance on trial two. Similar to the explanation provided above, it is possible that the different responses between the two trials was driven by a compulsion to respond in a fair manner across the two trials, although future research should explore why this pattern did not change over development (as we saw in Study 1).

## Comparing responses on the rope game and wealth-matching task

In Study 1, we found no age-related changes in the relation between our two status tasks. In contrast (and as described in the main text), in Study 2, responses on the two different status tasks showed greater convergence with age, $\beta$ = 1.3, *SE* = 0.43, *z* = 3, *p* = 0.003 (Fig SOM3).

## Analyses focusing on Black and White participants

### Black and White children’s use of race and gender as cues to social status

When only Black (*n* = 17) and White (*n* = 62) participants were included in analyses of Study 2, we found no main or interactive effects of participant race, target status, or age on participants responses on the rope task (all 95% Confidence Intervals (CI) include zero). Similarly, on the wealth-matching task we found no main or interactive effects of participant race or age (all *p*s > .10).

**The consequences of status** **beliefs in Black and White children**

*Status beliefs as measured by the rope task.* When we examined White and Black participants in Study 2, we found the same age-related changes as described in the main text, $\beta$ = 1.21, *SE* = 0.43, *z* = 2.82, *p* = .005, such that the probability of choosing a White affiliation partner increased with age. We found no significant effect of participant race on social partner preference (*p* > .10). Overall, Black participants did not choose Black social partners above chance, and were equally likely to select a Black as a White affiliation partner regardless of response type on the rope game (Black participants who responded in a stereotypical manner: *M* = 0.7, *SE* = 0.6; Black participants who responded in a counter-stereotypical manner: *M* = 0.68, *SE* = 0.5).

*Status beliefs as measured by the wealth-matching task.* When we examined Black and White participants only, we again found no main effect of response type on the wealth-matching task (*p* > .10). In this sample of participants, there was an effect of participant race, such that White participants were more likely than Black participants to select a White social partner, $\beta$ = -1.2, *SE* = 0.59, *z* = -2.04, *p* = 0.041. However, as noted above, Black participants were not above chance in selecting a Black social partner. Apart from the main effect of age (reported above and in the main text), there were no other main or interactive effects (all *p*s > .10).

## Subjective status in Black and White children

As before, there was a main effect of response type, $\beta$ = -0.61, *SE* = 0.3, *t* = -2.01 (95% CI = -1.17, -0.04), participants who responded in a counter-stereotypical manner rated themselves as higher in status than those who responded in a stereotypical manner. In contrast to the analyses reported in the main text, there were no other main or interactive effects in these analyses where only Black and White participants were included.

# Supplemental discussion

In sum, the patterns found in these supplemental analyses that focused on Black and White participants mimic many of the patterns described in the main text. However, they also illustrate the presence of in-group bias in Black participants, which shift some of their social preference responses further from the pro-White bias clearly evidenced by our White participants (a bias also present in the larger racial-ethnic minority participant sample). The small number of Black participants makes it difficult to draw any strong conclusions about this population, but illustrate the importance of including diverse groups of participants, and of exploring the role of participant social group membership on the development and consequences of status beliefs.

**Figures**


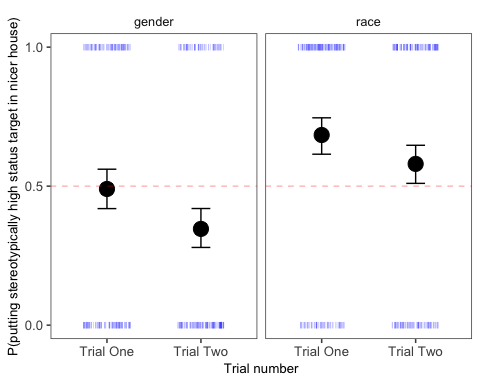


*Fig SOM1. Probability of placing the high-status target in the nicer house, as a function of trial number and social dimension (Study 1: gender; Study 2: race). The dotted line represents chance responding.* *Circles represent the means for each group, and error bars represent 95% confidence intervals around the means; dashes represent individual participants.*


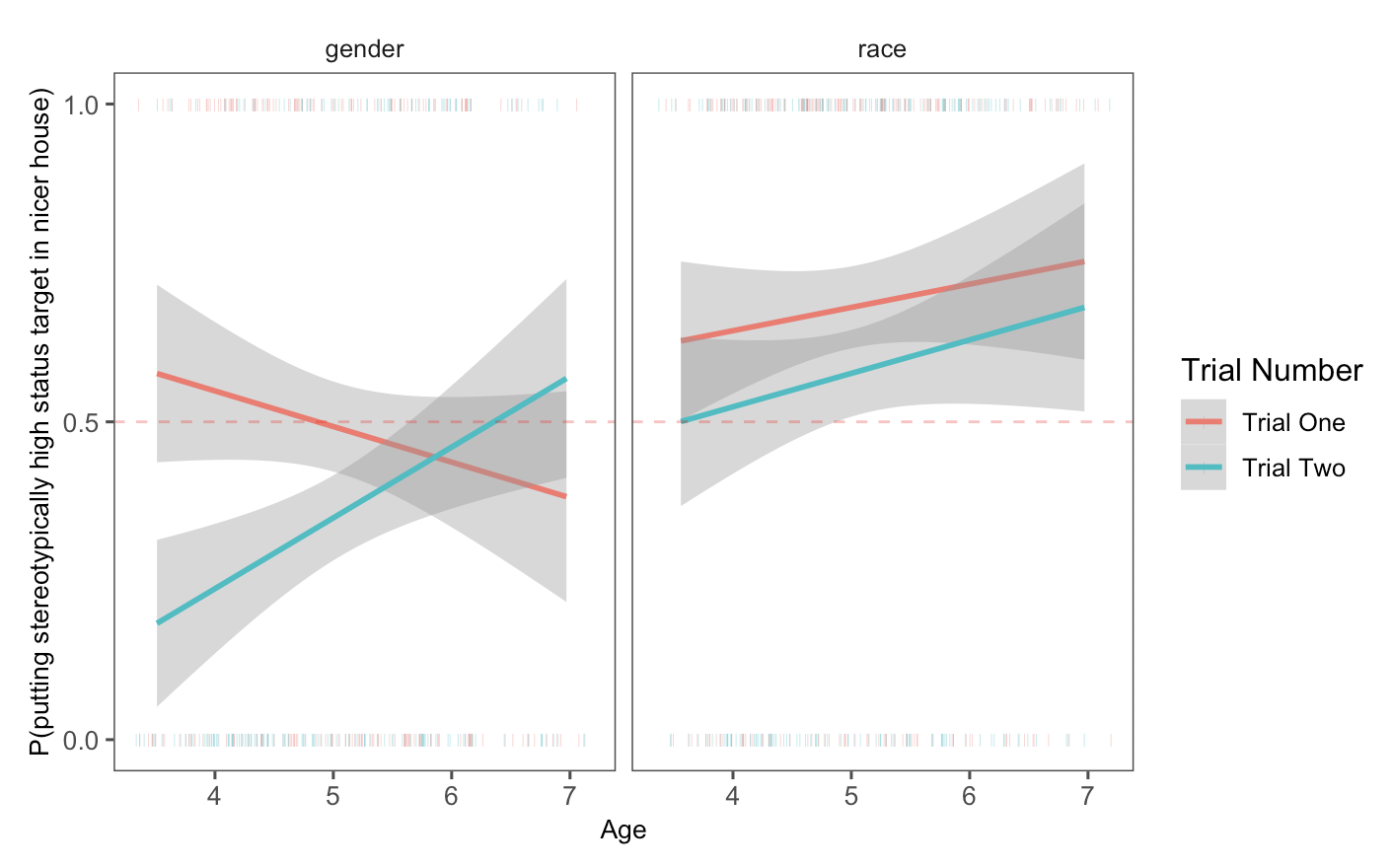


*Fig SOM2. Probability of placing the high-status target in the nicer house, as a function of age, trial number, and social dimension (Study 1: gender; Study 2: race). The dotted red line indicates chance responding. Shaded areas show 95% confidence bands around the regression lines; dashes represent individual participants.*


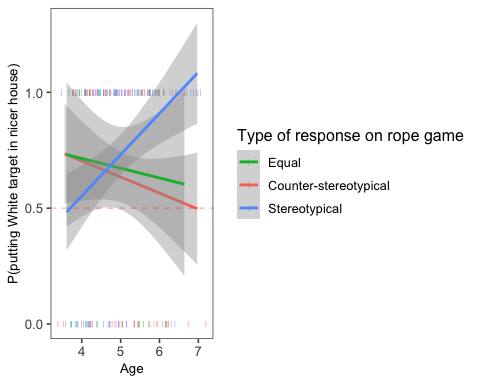


Fig SOM3. Probability of putting the high-status (White) target in the nicer house as a function of response type on the rope game and age. The dotted line represents chance responding. Shaded areas show 95% confidence bands around the regression lines; dashes represent individual participants.
