# Supplementary material for: Children's use of race and gender as cues to social status
Source: PLoS One. 2020 Jun 22;15(6):e0234398. doi: 10.1371/journal.pone.0234398 (PMC7307787; doi:10.1371/journal.pone.0234398)
Supplement: S1 Data — (DOCX) [file pone.0234398.s002.docx]

Repository data for “Children’s use of race and gender as cues to social status”

Open Science Framework (data, analysis code, some study materials): <https://osf.io/29vcu/>

Databrary (all materials, including protected images): <https://nyu.databrary.org/volume/599>
